# Destigmatizing Technology for Forming Safe Mental Illness Disclosure Skills for Patients with Endogenous Mental Disorders

**DOI:** 10.1192/j.eurpsy.2026.10980

**Published:** 2026-07-31

**Authors:** V. Mitikhin, G. Tiumenkova

**Affiliations:** Mental Health Research Centre, Moscow, Russian Federation

## Abstract

**Introduction:**

As research results show, patients with endogenous mental disorders already at an early stage of the disease show an increased level of self-stigmatization (Thornicroft G., et al., 2022; T. Solokhina et al., 2023; 2024). A number of destigmatization approaches are presented in foreign literature (Setti et al., 2019; Dubreucq J., et al., 2021), however, in our country, technologies aimed directly at working with patients practically do not exist, which determines the relevance of their development.

**Objectives:**

Development of organizational and methodological approaches to conducting destigmatization training to develop skills for safe communication about one’s mental illness.

**Methods:**

22 training participants were patients at the stage of the first psychotic episode with schizophrenia spectrum disorders (F20, F23, F25 according to ICD-10) and with a diagnosis of bipolar disorder (F31.xxx according to ICD-10). The duration of the disease ranged from 6 months to 3 years.

**Results:**

When creating the organizational and methodological approaches for the training “Formation of skills for safe reporting of one’s mental illness”, the experience of foreign researchers was used (P.W. Corrigan, B.A. Buchholz, “Coming Out Proud” (2013). Developed and adapted, considering cultural characteristics, the training was based on a multimodal approach, including psychoeducation, elements of cognitive-behavioral, emotive-reflexive and narrative therapy. The technology was a short-term intervention, which was conducted in a closed group, included 4 psychoeducational meetings and 3 thematic sessions. During psychoeducation, the emphasis was on overcoming stigma, expanding knowledge about mental illnesses. The main topics of the sessions for discussion were determined in accordance with the P.W. Corrigan program: “Considering the pros and cons of disclosing information”; “Discussing various ways of disclosure”; “Telling your story”. The sessions were held in an interactive format. Participants, training together with the leaders considered tasks, the solution of which allowed them to go beyond their own limitations, to gain new knowledge and skills. During the training, the participants learned to talk about themselves, their experiences, to share experiences, which contributed to a decrease in anxiety, an improvement in mood, and an increase in self-esteem.

**Conclusions:**

Technology has shown its potential, however, for further implementation into practice and inclusion in psychosocial rehabilitation programs, an assessment of its effectiveness is necessary, which will be the next stage of our research.

**Disclosure of Interest:**

None Declared

